# Health literacy and recurrence risk perception among stroke patients: insights from a cognitive resource perspective

**DOI:** 10.3389/fpubh.2026.1868971

**Published:** 2026-08-06

**Authors:** Ling Yuan, Yuanmei Zhao, Chunyu He, Yue Wang, Xiaoqing Jiang, Yurou Tian, Zhen Zhuo

**Affiliations:** 1 School of Nursing, Chengdu Medical College, Chengdu, Sichuan, China; 2 The First Affiliated Hospital of Chengdu Medical College, Chengdu, Sichuan, China

**Keywords:** coping styles, health literacy, public health, risk perception, secondary prevention, social support, stroke

## Abstract

**Objective:**

To examine the association between health literacy and recurrence risk perception among stroke patients and to explore the relationships of social support and coping styles with recurrence risk perception.

**Methods:**

A cross-sectional study was conducted among 200 young and middle-aged stroke patients recruited from three tertiary hospitals in Chengdu, China, between December 2025 and April 2026. Data were collected using the Health Literacy Scale for Stroke Patients (HLSSP), Stroke Recurrence Risk Perception Scale, Social Support Rating Scale (SSRS), and Medical Coping Modes Questionnaire (MCMQ). Pearson correlation, multiple linear regression, and mediation analysis using the PROCESS macro (Model 4) were performed.

**Results:**

Health literacy was positively correlated with recurrence risk perception (r = 0.397, *P* < 0.01) and social support (r = 0.269, *P* < 0.01). In multivariable regression analyses, both health literacy (β = 0.169, *P* = 0.013) and social support (β = 0.168, *P* = 0.008) were significant positive predictors after adjusting for demographic variables. Age was negatively associated (β = −0.205, *P* = 0.002), whereas education level showed a positive association (β = 0.286, *P* < 0.001). The model explained 32.6% of the variance. Mediation analysis indicated that coping styles did not have significant indirect effects (95% bootstrap CI included zero).

**Conclusion:**

Health literacy and social support are key determinants of recurrence risk perception among stroke patients. The findings are consistent with a cognitive resource perspective and suggest that health literacy and social support may be associated with recurrence risk perception among stroke patients. Further longitudinal studies are needed to clarify the underlying mechanisms. Coping styles did not mediate this relationship. These results highlight the importance of integrating health literacy and social support interventions into community-based stroke management to promote appropriate risk perception and secondary prevention.

## 1 Introduction

Stroke remains one of the leading causes of disability and mortality worldwide, with a continuously increasing disease burden. Beyond motor and neurological impairments, stroke has profound and long-term impacts on patients' social role adaptation and psychological wellbeing. According to recent reports, stroke is a major contributor to global disability and substantially reduces quality of life (1). Globally, stroke remains one of the leading causes of death and long-term disability, with an estimated 12.2 million new cases and 6.6 million deaths annually (2). In China, the burden is disproportionately high, with approximately 4 million new stroke cases each year and a prevalence of over 13 million, making stroke the leading cause of death among Chinese adults (3). Within Sichuan Province, recent epidemiological data indicate that the stroke incidence rate was approximately 500.28 per 100,000 population in 2024, with an estimated 50,000 new cases occurring annually in Chengdu alone (4). During the prolonged rehabilitation process, patients must continuously adjust to changes in their family and social roles, which is often accompanied by persistent concern about disease recurrence. Consequently, recurrence risk perception has emerged as an important psychological factor influencing rehabilitation outcomes and secondary prevention behaviors (5). Previous studies have demonstrated that patients' perceptions of disease risk are closely associated with their health behaviors and psychological adaptation (6, 7). However, existing research has primarily focused on functional recovery or isolated psychological outcomes, while the determinants and underlying mechanisms of recurrence risk perception remain insufficiently understood.

Health literacy, defined as the ability to obtain, understand, and apply health-related information to make appropriate health decisions, is widely recognized as a key determinant in chronic disease management (8). Higher levels of health literacy have been consistently associated with improved health behaviors, better disease management, and more favorable health outcomes (8, 9). In stroke and other chronic disease populations, health literacy is not only linked to self-management behaviors but also to psychological outcomes such as depression and anxiety (10, 11). Emerging evidence suggests that health literacy may influence individuals' cognitive processing of health information, thereby shaping risk perception and decision-making processes (8, 12). From this perspective, health literacy may function as a core cognitive resource that enhances individuals' capacity to appraise disease-related risks. Nevertheless, the specific pathways through which health literacy affects recurrence risk perception remain unclear, and the relationship may be complex and potentially non-linear.

From a theoretical perspective, the stress–coping model posits that individuals respond to disease-related stress through cognitive appraisal and coping strategies, which subsequently influence psychological outcomes (13). Within this framework, coping styles are often considered key mechanisms linking cognitive factors to psychological responses. However, empirical findings regarding the roles of different coping strategies—such as confrontation, avoidance, and resignation—have been inconsistent. In addition, social support, as a critical external resource, plays a fundamental role in the adaptation process. Beyond its traditional buffering effect on stress, social support may facilitate access to health information and enhance individuals' cognitive and behavioral responses through informational and emotional pathways (14). Evidence from stroke populations also highlights the importance of social support in recovery and adaptation (15). Despite these insights, the potential roles of coping styles and social support in the relationship between health literacy and risk perception remain inconclusive, particularly in stroke patients.

**Figure 1:**
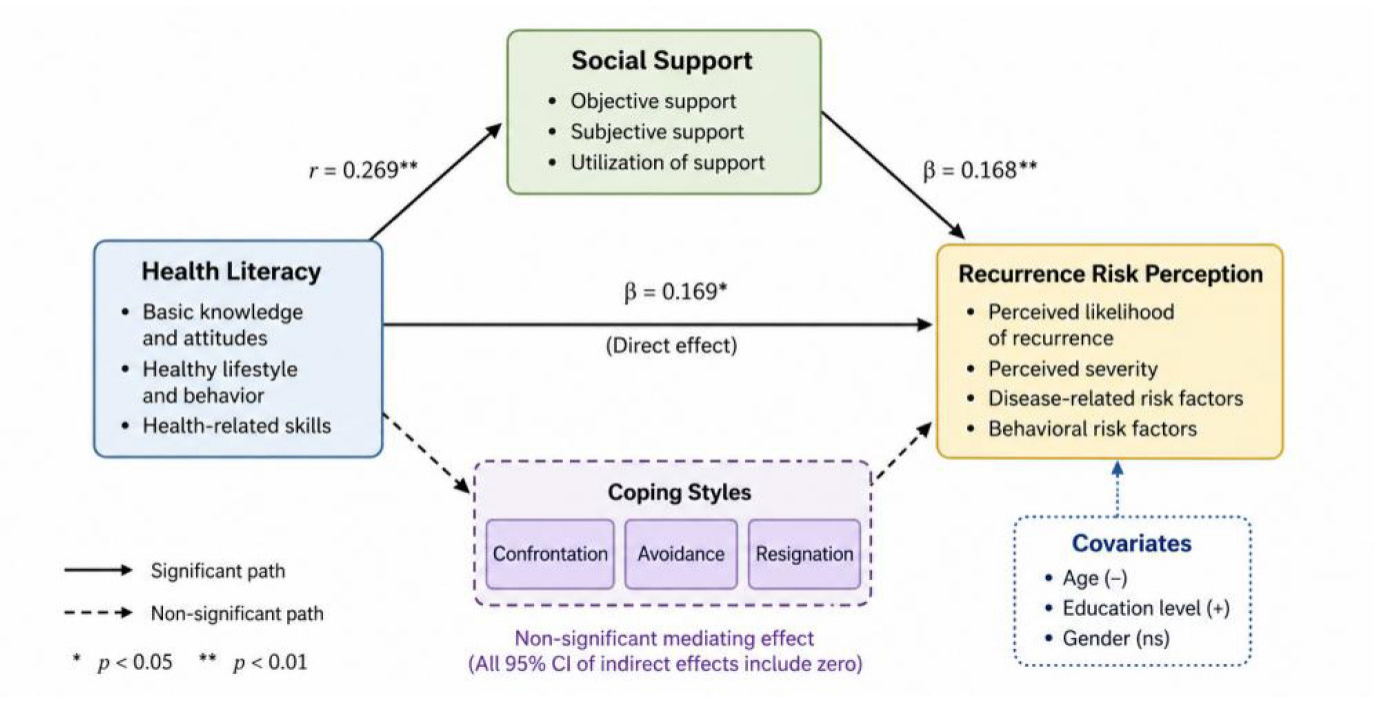
Conceptual framework of the study.

Therefore, this study focused on young and middle-aged stroke patients to examine the association between health literacy and recurrence risk perception after adjusting for key demographic variables. In addition, the potential roles of coping styles and social support were explored within a theoretical framework. From a public health perspective, elucidating these relationships may inform the development of targeted interventions to improve risk awareness and optimize secondary prevention strategies in stroke populations. Based on the theoretical framework, a conceptual model was developed (Figure 1).

This figure illustrates the hypothesized relationships among health literacy, social support, coping styles, and recurrence risk perception. Health literacy is proposed as a core cognitive resource influencing risk perception directly, while social support functions as an external facilitating factor. Coping styles were hypothesized as potential mediators.

## 2 Methods

### 2.1 Study design and participants

A cross-sectional study was conducted between December 2025 and April 2026. Participants were recruited using convenience sampling from the neurology and rehabilitation departments of three tertiary hospitals in Chengdu, China. Potential participants were initially identified through medical record screening. Patients who met the predefined inclusion and exclusion criteria were approached by trained researchers during hospitalization and invited to participate in the study. Written informed consent was obtained prior to questionnaire administration.

The inclusion criteria were: (1) diagnosis of stroke confirmed by computed tomography (CT) or magnetic resonance imaging (MRI); (2) age between 18 and 59 years; (3) clear consciousness, no communication barriers, stable condition, and ability to complete the questionnaire; and (4) provision of informed consent.

The exclusion criteria were: (1) unstable medical condition or severe comorbidities (e.g., serious cardiac or renal diseases); (2) severe cognitive impairment or psychiatric disorders; and (3) inability or unwillingness to complete the questionnaire.

**Figure 2:**
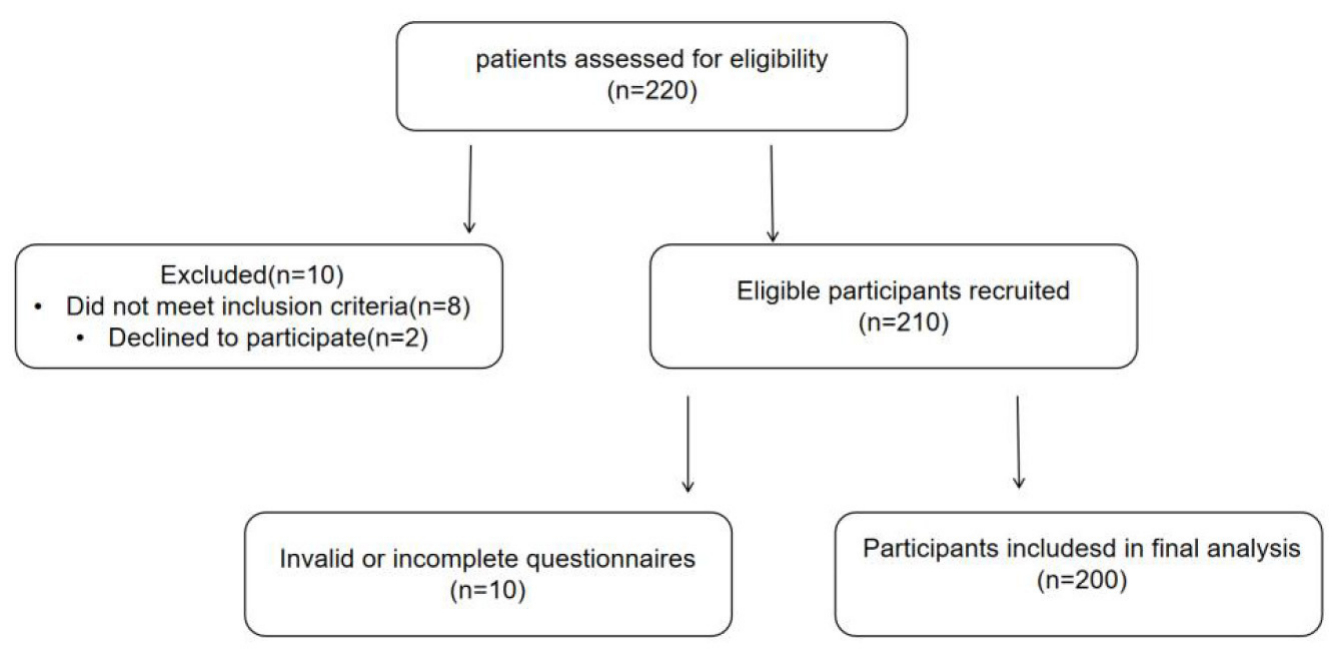
Participant recruitment and selection.

A total of 210 questionnaires were distributed, and 200 valid responses were obtained, yielding a response rate of 95.24% (Figure 2).

### 2.2 Measures

#### 2.2.1 Demographic characteristics

Demographic information was collected using a self-designed questionnaire, including age, gender, marital status, educational level, and monthly household income.

#### 2.2.2 Recurrence risk perception

Recurrence risk perception was assessed using the Stroke Recurrence Risk Perception Scale developed by Lin et al (16). The scale consists of two parts: perceived likelihood of recurrence (3 items), and a multidimensional assessment including perceived severity (7 items), disease-related risk factors (6 items), and behavioral risk factors (4 items), totaling 17 items.

The score was calculated as the mean of all item scores, with higher scores indicating higher levels of perceived recurrence risk. In this study, the scale demonstrated good internal consistency, with a Cronbach's **α** of 0.850 for the second part and 0.875, 0.815, and 0.804 for its subdimensions.

#### 2.2.3 Health literacy

Health literacy was measured using the Health Literacy Scale for Stroke Patients (HLSSP) developed by Liu et al (17). The scale includes three dimensions: basic knowledge and attitudes, healthy lifestyle and behavior, and health-related skills, comprising 20 items rated on a 5-point Likert scale. The total score was calculated as the mean of all item scores (range 1–5), with higher scores indicating higher levels of health literacy. The Cronbach's α coefficient in this study was 0.929.

#### 2.2.4 Social support

Social support was assessed using the Social Support Rating Scale (SSRS) (18). The scale consists of three dimensions: objective support, subjective support, and utilization of support, with a total of 10 items. Scores range from 12 to 66, with higher scores indicating higher levels of social support. The Cronbach's **α** coefficient in this study was 0.72.

#### 2.2.5 Coping styles

Coping styles were assessed using the Medical Coping Modes Questionnaire (MCMQ) (19). The scale includes three dimensions: confrontation, avoidance, and resignation, with a total of 20 items rated on a 4-point Likert scale. Higher scores indicate a stronger tendency toward a specific coping style. The Cronbach's **α** coefficients for the three dimensions in this study were 0.69, 0.60, and 0.76, respectively.

### 2.3 Sample size

The sample size was estimated based on the rule of thumb recommended by Kendall, which suggests that the sample size should be 5 to 10 times the number of independent variables to be included in the analysis. This approach is widely used in exploratory cross-sectional studies in health and nursing research. A total of 16 variables were included; therefore, the minimum required sample size was 160. Considering a potential 20% invalid response rate, the target sample size was adjusted to at least 192. Ultimately, 200 participants were included, meeting the requirements for statistical analysis.

### 2.4 Data collection

Eligible patients were screened according to the predefined inclusion and exclusion criteria and invited to participate during hospitalization by trained researchers. All questionnaires were self-administered. For participants with reading or writing difficulties, trained researchers read the items aloud and recorded their responses. Data were collected through face-to-face surveys conducted by trained researchers. Participants were informed about the study purpose and procedures prior to participation, and written informed consent was obtained. All questionnaires were collected on site and checked for completeness and logical consistency to ensure data quality.

### 2.5 Statistical analysis

All statistical analyses were performed using SPSS version 26.0. Continuous variables were expressed as mean ± standard deviation (Mean ± SD).

Pearson correlation analysis was used to examine the relationships among study variables. Multiple linear regression analysis was conducted with recurrence risk perception as the dependent variable, health literacy and social support as the main independent variables, and age, gender, and educational level as covariates.

Multicollinearity was assessed using variance inflation factors (VIF), with VIF values < 5 were considered indicative of no significant multicollinearity.

Mediation analysis was performed using the PROCESS macro (Model 4) to examine the potential mediating role of coping styles in the relationship between health literacy and recurrence risk perception. A bootstrap method with 5,000 resamples was applied to estimate 95% confidence intervals (CI). Indirect effects were considered statistically significant if the confidence intervals did not include zero.

In addition, assumptions of linear regression, including normality and homoscedasticity of residuals, were examined. Continuous variables were centered when necessary to improve model stability. All statistical tests were two-tailed, with a significance level set at *P* < 0.05.

### 2.6 Ethics statement

This study was approved by the Institutional Ethics Committee (Approval No. CMCIR2025NO.062). All participants provided written informed consent prior to participation.

## 3 Results

### 3.1 Descriptive statistics of study variables

A total of 200 stroke patients were included in the analysis. Participant characteristics are presented in Table 1, and descriptive statistics of the study variables are presented in Table 2.

**Table 1:** Participant characteristics (*n* = 200).

Variable	Category	*n* (%)/mean ±SD
Age (years)		52.98 ± 6.17
Gender	Male	122 (61.0)
	Female	78 (39.0)
Marital status	Married	186 (93.0)
	Unmarried	4 (2.0)
	Others (widowed/divorced)	10 (5.0)
Education level	Low (primary school & junior high school)	151 (75.5)
	Medium (high school)	30 (15.0)
	High (junior college & above)	19 (9.5)
Occupation	Farmer	53 (26.5)
	Retired	9 (4.5)
	Employee	84 (42.0)
	Others	54 (27.0)
Income	1,001–3,000	62 (31.0)
	≥3,001	138 (69.0)
Payment method	Urban employee basic medical insurance	103 (51.5)
	Urban and rural resident basic medical insurance	51 (25.5)
	Others (self-pay/commercial insurance, etc.)	46 (23.0)

Values are presented as mean ± standard deviation (SD) or number (percentage).

**Table 2:** Descriptive statistics of study variables (*n* = 200).

Variable	Min	Max	Mean	SD
HL	2.35	4.43	3.14	0.34
RP	1.82	8.00	4.32	1.86
F (confrontation)	8	16	12.15	1.77
A (avoidance)	15	28	20.52	2.44
Y (resignation)	13	22	18.44	1.70
SSRS	22	56	37.88	4.92

HL, health literacy; RP, recurrence risk perception; F, confrontation; A, avoidance; Y, resignation; SSRS, Social Support Rating Scale.

The mean health literacy score was (3.14 ± 0.34), indicating a moderate level. The mean perceived risk of recurrence was (4.32 ± 1.86), suggesting considerable inter-individual variability.

Regarding coping strategies, avoidance (20.52 ± 2.44) and resignation (18.44 ± 1.70) scores were higher than confrontation (12.15 ± 1.77), indicating a tendency toward more passive coping styles. The overall social support score was 37.88 ± 4.92, reflecting a moderate level.

### 3.2 Correlation analysis

**Figure 3:**
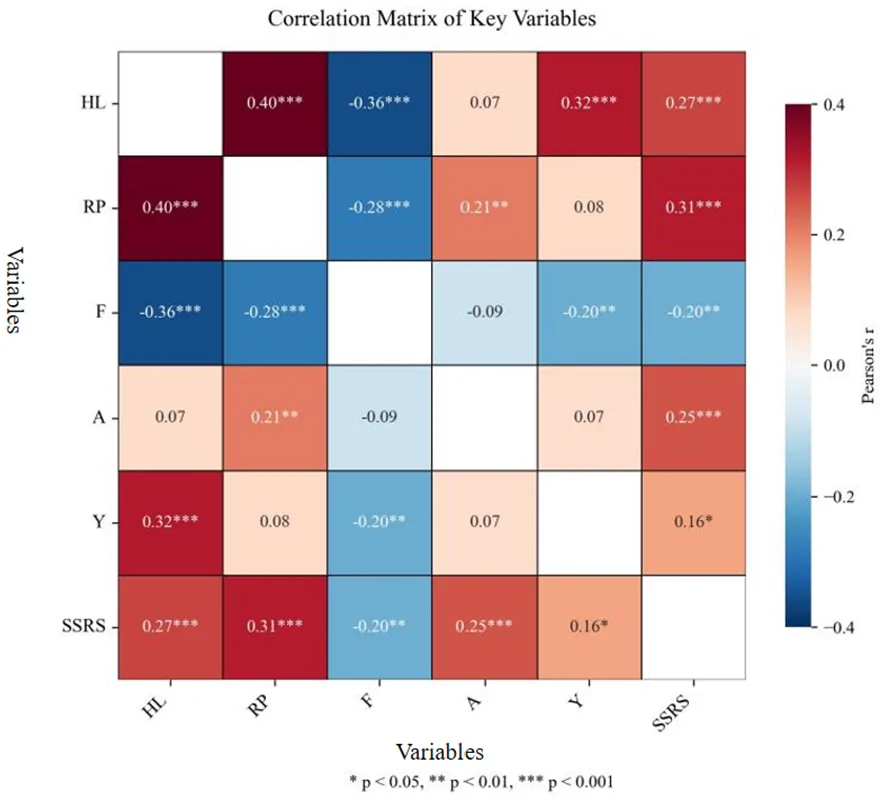
Correlation heatmap of study variables. The color intensity represents the strength and direction of correlations. Red indicates positive correlations and blue indicates negative correlations. HL, health literacy; RP, recurrence risk perception; F, confrontation; A, avoidance; Y, resignation; SSRS, social support.

Pearson correlation coefficients are presented in Table 3 and visualized in Figure 3.

**Table 3:** Correlations among study variables (*n* = 200).

Variable	Mean ±SD	1	2	3	4	5	6
1 HL	3.14 ± 0.34	1					
2 RP	4.32 ± 1.86	0.397^**^	1				
3 F (confrontation)	12.15 ± 1.77	−0.358^**^	−0.283^**^	1			
4 A (avoidance)	20.52 ± 2.44	0.075	0.207^**^	−0.087	1		
5 Y (resignation)	18.44 ± 1.70	0.317^**^	0.079	−0.197^**^	0.072	1	
6 SSRS	37.88 ± 4.92	0.269^**^	0.313^**^	−0.195^**^	0.253^**^	0.160^*^	1

HL, health literacy; RP, recurrence risk perception; SSRS, Social Support Rating Scale. F, A, Y are subscales of the Medical Coping Modes Questionnaire (MCMQ).

^*^p < 0.05;

^**^p < 0.01.

Health literacy was moderately and positively correlated with perceived risk of recurrence (r = 0.397, *P* < 0.01), and also positively correlated with social support (r = 0.269, *P* < 0.01).

In terms of coping strategies, health literacy was negatively associated with confrontation (r = −0.358, *P* < 0.01) and positively associated with resignation (r = 0.317, *P* < 0.01), while its association with avoidance was not statistically significant.

Perceived risk of recurrence was negatively correlated with confrontation (r = −0.283, *P* < 0.01), and positively correlated with avoidance (r = 0.207, *P* < 0.01) and social support (r = 0.313, *P* < 0.01).

Overall, the correlation coefficients ranged from small to moderate (|r| ≈ 0.20–0.40), suggesting meaningful but not excessive associations among variables.

The correlation heatmap (Figure 3) showed a pattern consistent with the numerical results, highlighting distinct directional relationships among coping dimensions and key variables.

### 3.3 Multiple linear regression analysis

The results of the multiple linear regression analysis are presented in Table 4.

**Table 4:** Multiple linear regression analysis of recurrence risk perception (*n* = 200).

Variable	B	SE	β	*t*	P	95% CI
Constant	1.335	1.885	—	0.708	0.480	−2.383 5.053
HL	0.934	0.373	0.169	2.507	0.013	0.199 1.670
SSRS	0.063	0.024	0.168	2.681	0.008	0.017 0.110
Age	−0.062	0.019	−0.205	−3.200	0.002	−0.100 −0.024
Gender	−0.047	0.232	−0.012	−0.201	0.841	−0.504 0.411
Education	0.495	0.117	0.286	4.237	< 0.001	0.265 0.726

β indicates standardized coefficients. R^2^ = 0.326, F = 18.810, *P* < 0.001. No multicollinearity was detected (all VIF < 2).

After adjusting for age, gender, and education level, health literacy remained a significant positive predictor of perceived risk of recurrence (B = 0.934, β = 0.169, *P* = 0.013). Social support was also significantly associated with the outcome (B = 0.063, **β** = 0.168, *P* = 0.008).

Among the covariates, age was negatively associated with perceived risk of recurrence (B = −0.062, **β** **=** **–**0.205, *P* = 0.002), whereas education level showed a positive association (B = 0.495, **β = ** 0.286, *P* < 0.001). Gender was not significantly associated with the outcome (*P* > 0.05).

The overall model was statistically significant (F = 18.810, *P* < 0.001), explaining 32.6% of the variance in perceived risk of recurrence (R^2^ = 0.326).

**Figure 4:**
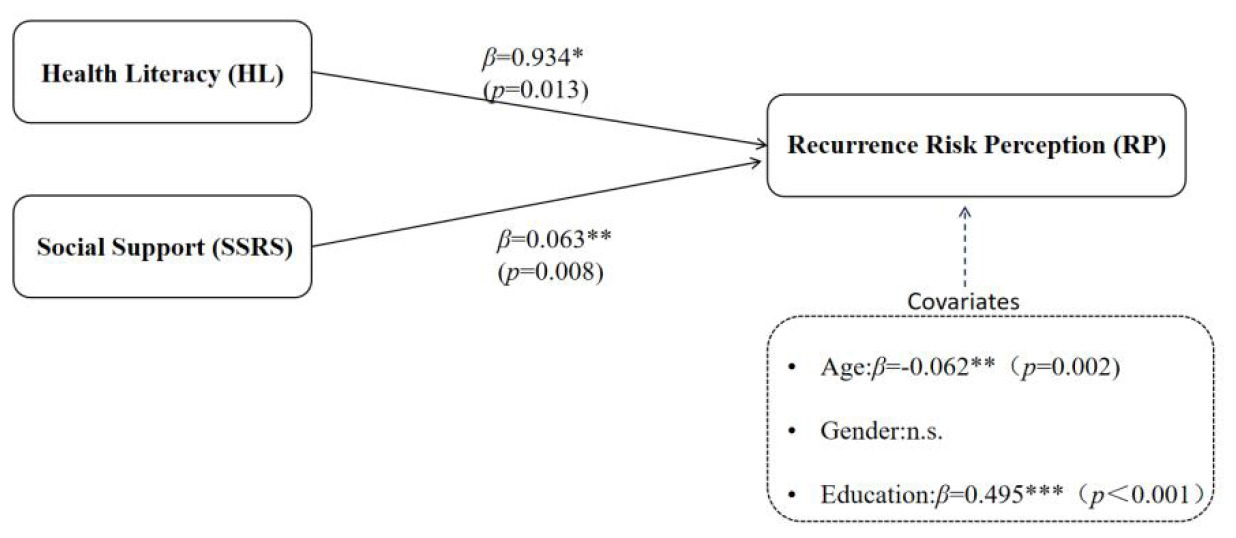
Association between health literacy and recurrence risk perception. The solid line represents the predicted values from the adjusted regression model, and the shaded area indicates the 95% confidence interval. The model was adjusted for age, gender, and education level. The fitted regression line indicates a positive linear association between health literacy and recurrence risk perception after adjusting for covariates. The shaded area represents the 95% confidence interval.

No evidence of multicollinearity was detected, as all variance inflation factors (VIFs) were below 2. Figure 4 illustrates the marginal effect of health literacy on perceived risk of recurrence, demonstrating a stable positive linear relationship across the observed range. The VIF values for all independent variables ranged from 1.071 to 1.317 (SSRS = 1.128, HL = 1.312, age = 1.182, gender = 1.071, education = 1.317), indicating no significant multicollinearity.

### 3.4 Mediation analysis

Exploratory mediation analysis was conducted using the PROCESS macro (Model 4). The indirect effects of coping strategies were not statistically significant, as the 95% bootstrap confidence intervals included zero, indicating that coping styles did not mediate the relationship between health literacy and perceived risk of recurrence.

## 4 Discussion

### 4.1 Principal findings

This study examined the relationships among health literacy, social support, coping styles, and recurrence risk perception in young and middle-aged stroke patients. The findings indicate that health literacy was positively associated with recurrence risk perception, and social support also showed a significant positive association, whereas coping styles did not demonstrate a significant mediating effect. Overall, these findings are consistent with a cognitive resource perspective and suggest that health literacy and social support may be associated with recurrence risk perception. However, the hypothesized mediating role of coping styles was not supported, indicating that existing theoretical assumptions may operate differently in this specific context.

### 4.2 Role of health literacy in shaping risk perception

Health literacy was identified as a significant positive predictor of recurrence risk perception, a finding consistently supported by both correlation and regression analyses. Previous studies have shown that individuals with higher health literacy are better able to obtain, understand, and apply health information, thereby enabling more accurate risk appraisal and decision-making (8, 12, 20). This capability is particularly important for stroke patients, as it facilitates a clearer understanding of recurrence risks and related factors (21).

Notably, the present findings suggest that health literacy may not only promote health behaviors but also enhance sensitivity to disease-related risks. This extends the traditional view that health literacy primarily influences behavioral outcomes and highlights its critical role in shaping disease-related cognition (8, 12). To further elaborate on this role, the three dimensions of the HLSSP may contribute to risk perception through distinct pathways (8). The knowledge and attitudes dimension equips patients with factual understanding of stroke pathophysiology and modifiable risk factors, which directly informs their perceived susceptibility to recurrence. The healthy lifestyle and behavior dimension reinforces awareness of actionable preventive measures, which may increase patients' sense of personal relevance regarding recurrence prevention. The health-related skills dimension enables patients to actively seek, interpret, and apply health information from various sources, thereby fostering a more accurate and comprehensive appraisal of their own recurrence risk.

### 4.3 Contribution of social support as a cognitive facilitator

Social support was also found to be a significant positive predictor of recurrence risk perception. Unlike the traditional “buffering” model, which emphasizes its role in stress reduction, the present findings suggest that social support may play a facilitating role in shaping risk perception (22). Specifically, social support may enhance access to health information and healthcare resources, while also providing informational and emotional support that improves patients' ability to interpret and evaluate disease-related risks (15, 23).

These findings indicate that, in stroke populations, social support may contribute to risk perception primarily through cognitive pathways rather than solely through stress reduction mechanisms (15). Regarding the specific types and sources of social support, the SSRS assesses three dimensions: objective support, subjective support, and support utilization. In this study, subjective support—particularly perceived emotional support from family members—appeared to be the strongest correlate of recurrence risk perception. This finding is consistent with previous research suggesting that emotional support from close relatives plays a central role in shaping illness perceptions in Chinese patient populations (14, 15). Informational support from healthcare providers also contributed to patients' understanding of recurrence risks, whereas instrumental support showed a relatively weaker association. These results suggest that interventions aimed at enhancing risk perception should prioritize strengthening patients' perceived emotional support networks, particularly within the family context.

### 4.4 Lack of mediating effect of coping styles

No significant mediating effect of coping styles was observed in the relationship between health literacy and recurrence risk perception. Although certain coping dimensions were associated with the outcome in bivariate analyses, these associations did not remain significant after adjusting for other variables. This suggests that coping styles may not represent a primary pathway linking health literacy to risk perception.

From a mechanistic perspective, coping is inherently multidimensional and context-dependent (24). In the Chinese cultural context, several factors may shape patients' coping behaviors and their perception of social support. Traditional Chinese values, such as emotional restraint, acceptance of fate, and strong reliance on family networks, may influence how stroke patients interpret and respond to their illness^.^ Specifically, these cultural norms may predispose patients toward resignation coping, as accepting one's condition is often viewed as a form of emotional regulation rather than passivity. This may partly explain the positive association between health literacy and resignation coping observed in our sample: patients with greater disease understanding may adopt a more realistic and accepting attitude toward long-term stroke management, which is culturally congruent with the value of emotional equilibrium. In addition, the central role of the family in Chinese society means that social support—particularly from family members—is not only an external resource but also an integral part of patients' daily experience. This cultural embeddedness of family support may amplify its impact on risk perception, as patients are more likely to internalize family members' views and concerns about recurrence. These cultural considerations provide important context for interpreting the associations between social support, coping, and recurrence risk perception in this population. Interestingly, health literacy was negatively associated with confrontation coping and positively associated with resignation coping in this study. These findings differ from those reported in some previous studies and therefore warrant cautious interpretation. One possible explanation is that the confrontation dimension of the Chinese version of the MCMQ may reflect a more disease-focused or emotionally reactive response rather than an active information-seeking strategy. In addition, patients with higher health literacy may develop a more realistic understanding of the long-term nature of stroke management, which could influence their coping responses. Cultural factors may also shape the meaning and expression of coping behaviors in this population. Furthermore, the internal consistency of the confrontation dimension was relatively modest in the present study (Cronbach's α = 0.69). Therefore, these findings should be interpreted cautiously and require replication in larger and more diverse samples.

### 4.5 Integrated interpretation: a cognitive resource perspective

Taken together, the findings are consistent with a cognitive resource perspective in which health literacy may be associated with individuals' ability to understand and use health information, which may in turn relate to their perception of disease-related risks (25). Social support may serve as an external resource associated with greater access to and utilization of health information. In contrast, coping styles may play a more prominent role in emotional regulation and behavioral adaptation rather than directly influencing risk perception.

This integrated perspective contributes to a growing body of literature emphasizing the combined roles of cognitive and social resources in shaping health-related outcomes (23). This finding may contribute to refining existing theoretical models by emphasizing the primacy of cognitive resources over coping mechanisms in shaping risk perception in chronic disease contexts. In addition, although the final regression model explained 32.6% of the variance in recurrence risk perception, a substantial proportion remained unexplained, suggesting that recurrence risk perception is likely influenced by additional clinical, psychological, and contextual factors not assessed in the present study. The standardized coefficients for health literacy and social support were statistically significant but relatively modest (β ≈ 0.17), indicating that these variables represent only part of a broader set of determinants of recurrence risk perception.

### 4.6 Clinical implications

From a clinical perspective, these findings have important implications. Health literacy should be prioritized as a key target for intervention, with structured education programs designed to enhance patients' understanding of disease-related risks. In addition, strengthening social support systems—particularly in terms of informational and resource support—may facilitate more accurate and comprehensive risk awareness. Although coping styles did not mediate the relationship between health literacy and risk perception, they remain clinically relevant for patients' emotional adjustment and behavioral adaptation during recovery. We suggest that clinicians routinely assess patients' coping styles during follow-up consultations, paying particular attention to passive or maladaptive patterns such as resignation and avoidance. While these coping styles may not directly alter risk perception, they can influence patients' engagement with rehabilitation and adherence to secondary prevention recommendations. Therefore, clinicians may consider providing brief psychological support or targeted counseling for patients who exhibit maladaptive coping, while continuing to prioritize health literacy enhancement and social support strengthening as primary intervention targets.

### 4.7 Public health implications

From a public health perspective, the findings underscore the importance of integrating health literacy interventions into community-based stroke management programs (26). Improving patients' capacity to access, understand, and apply health information may enhance risk awareness and promote secondary prevention. In addition, strengthening social support systems-particularly through family and community networks-may facilitate more effective dissemination and utilization of health information.

### 4.8 Limitations and future directions

Several limitations should be acknowledged. First, the cross-sectional design precludes causal inference. Second, the sample was drawn from a limited geographic region, which may restrict the generalizability of the findings. In addition, the use of convenience sampling may have introduced selection bias and may limit the representativeness of the study population. Third, the use of self-reported measures may introduce reporting bias. Fourth, important clinical characteristics, including stroke type, severity, time since stroke, and history of previous stroke, were not collected and therefore could not be controlled for in the analyses. These factors may influence recurrence risk perception and may partially account for the unexplained variance observed in the regression model. Future studies should incorporate these clinical characteristics to provide a more comprehensive understanding of recurrence risk perception.

Future studies using longitudinal designs and more advanced analytical approaches, such as structural equation modeling, are warranted to further elucidate causal relationships and underlying mechanisms.

## 5 Conclusion

The findings are consistent with a cognitive resource perspective and suggest that health literacy and social support may be associated with recurrence risk perception among stroke patients. These findings highlight the potential importance of health literacy and social support in stroke management and secondary prevention. Further longitudinal studies are needed to clarify the underlying mechanisms.

## Data Availability

The original contributions presented in the study are included in the article/supplementary material, further inquiries can be directed to the corresponding author.
